# Supplementary material for: Isolation and Comprehensive in Silico Characterisation of a New 3-Hydroxy-3-Methylglutaryl-Coenzyme A Reductase 4 (HMGR4) Gene Promoter from Salvia miltiorrhiza: Comparative Analyses of Plant HMGR Promoters
Source: Plants (Basel). 2022 Jul 16;11(14):1861. doi: 10.3390/plants11141861 (PMC9318348; doi:10.3390/plants11141861)
Supplement: Supplementary file 1 [file plants-11-01861-s001.zip › Figure S1.pdf]

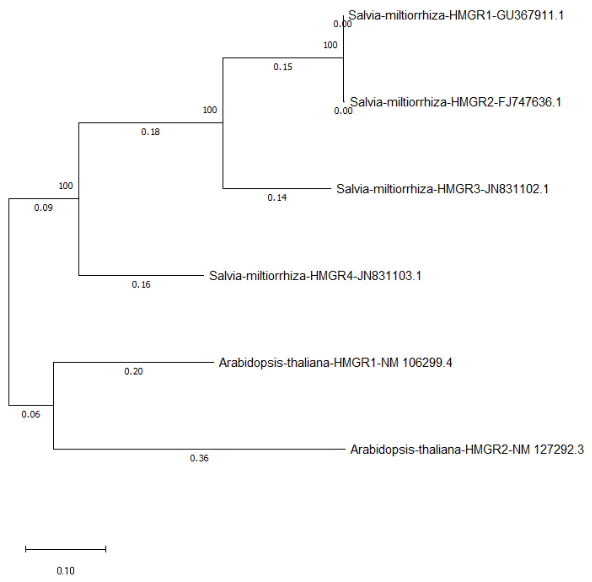

Figure S1. Unrooted dendrogram of *HMGR* sequences from *S. miltiorrhiza* and *A. thaliana* constructed by Maximum Likelihood method and bootstrap test with 1,000 replicates using the MEGA X program.
