# Supplementary material for: Isolation and Comprehensive in Silico Characterisation of a New 3-Hydroxy-3-Methylglutaryl-Coenzyme A Reductase 4 (HMGR4) Gene Promoter from Salvia miltiorrhiza: Comparative Analyses of Plant HMGR Promoters
Source: Plants (Basel). 2022 Jul 16;11(14):1861. doi: 10.3390/plants11141861 (PMC9318348; doi:10.3390/plants11141861)
Supplement: Supplementary file 1 [file plants-11-01861-s001.zip › Table S1.pdf]

Table S1. TFs responsive to light, hormone, wounding and pathogen stimulation found in the entire *S. miltiorrhiza HMGR4* promoter sequence using PlantPan 2.0 database.

TF family name	TF name
	**light-responsive TFs**
bHLH	BHLH63, PIF1
bZIP	BZIP54
Dehydrin	COR15A
Dof	DOF1.5, DOF1.10, DOF2.5, DOF3.3, DOF5.2
GATA	GATA1, GATA2, GATA3, GATA4, GATA5, GATA8, GATA9, GATA21, GATA22, GATA23
Homeodomain; bZIP; HD-ZIP	ATHB-1
Homeodomain; HD-ZIP	ATHB-2, ATHB-4, ATHB-16
MYB-related/Myb/SANT	RVE7
NAC; NAM	NAC081
	**auxin-responsive TFs**
bHLH	BEE1, BEE3
Dof	DOF1.1, DOF3.4, DOF3.6
Homeodomain; bZIP; HD-ZIP	ATHB-15
Homeodomain; HB-PHD	PRH
Homeodomain; HD-ZIP	ATHB-2, ATHB-20
MADS box ;MIKC	AGL14, AGL15
MYB-related	CCA1, RVE4, RVE5, RVE8
Myb/SANT; MYB	MYB6
NAC; NAM	NAC030, NAC101
WRKY	WRKY23
	**SA-responsive TFs**
bHLH	BHLH66
CAMTA	CAMTA2, CAMTA4, CAMTA6
CSD	CSP2
Dof	DOF1.1, DOF3.4, DOF3.6
MYB	MYB46
MYB-related	CCA1, RVE4, RVE5, RVE6, RVE8
Myb/SANT	MYB3, MYB6
NAC; NAM	NAC062, NAC081
WRKY	WRKY3, WRKY4, WRKY6, WRKY7, WRKY8, WRKY15, WRKY18, WRKY21, WRKY26, WRKY30, WRKY40, WRKY53, WRKY54, WRKY60, WRKY70
	**brassinosteroid-responsive TFs**
AP2; B3; RAV	RAV1
bHLH	BEE1, BEE3
NAC; NAM	NAC030, NAC081, NAC101
	**ethylene-responsive TFs**
AP2; ERF	RAP2-2
bHLH	BEE1, BEE3, BHLH66
C2H2	AZF2, AZF3
CAMTA	CAMTA1, CAMTA4
CG-1; CAMTA	CAMTA3
CSD	CSP2
MYB-related	CCA1, RVE4, RVE5, RVE6, RVE8
MYB-related/Myb/SANT	RVE7
Myb/SANT;ARR-B	ARR2
Myb/SANT; MYB	MYB6
NAC; NAM	NAC029
WRKY	WRKY4, WRKY6
	**ABA-responsive TFs**

bHLH	BEE1, BEE3, MYC2, SCRM
bZIP	ABF3, ABF4
C2H2	AZF2, AZF, ZAT10
CG-1; CAMTA	CAMTA2, CAMTA4, CAMTA6
CSD	CSP2
Dehydrin	COR15A
Homeodomain; HD-ZIP	HAT22
MYB-related	CCA1, RVE4, RVE5, RVE6, RVE8
Myb/SANT	MYB7
Myb/SANT; MYB	MYB3
NAC; NAM	NAC019, NAC029, NAC032, NAC055, NAC062, NAC072
WRKY	WRKY8
ZF-HD	ZHD5
**MeJa-responsive TFs**	
AP2; ERF	RAP2-3
bHLH	MYC2
CAMTA	CAMTA1, CAMTA4
CG-1; CAMTA	CAMTA3
NAC; NAM	NAC081
**wounding-responsive TFs**	
C2H2	ZAT10
CAMTA	CAMTA1, CAMTA4, CAMTA6
CG-1; CAMTA	CAMTA2, CAMTA3
NAC; NAM	NAC002, NAC081
WRKY	WRKY6, WRKY8, WRKY20, WRKY40
**pathogen-responsive TFs**	
CG-1; CAMTA	CAMTA3
Homeodomain; HB-PHD	PRH
Myb/SANT; MYB	MYB6
NAC; NAM	NAC081, NAC091
SBP	SPL14
WRKY	WRKY4, WRKY6, WRKY8, WRKY11, WRKY17, WRKY18, WRKY23, WRKY27, WRKY33, WRKY38, WRKY48, WRKY53, WRKY60
