# Supplementary material for: Isolation and Comprehensive in Silico Characterisation of a New 3-Hydroxy-3-Methylglutaryl-Coenzyme A Reductase 4 (HMGR4) Gene Promoter from Salvia miltiorrhiza: Comparative Analyses of Plant HMGR Promoters
Source: Plants (Basel). 2022 Jul 16;11(14):1861. doi: 10.3390/plants11141861 (PMC9318348; doi:10.3390/plants11141861)
Supplement: Supplementary file 1 [file plants-11-01861-s001.zip › Table S2.pdf]

Table S2. TFs responsive to major abiotic and biotic factors found in the proximal *S. miltiorrhiza HMGR4* promoter using the PlantPan 2.0 database.

TF family name	TF name
	**light-responsive TFs**
bHLH	MYC2
Dehydrin	COR15A
Dof	DOF1.5, DOF2.5
GATA	GATA1, GATA2, GATA3, GATA4, GATA5, GATA8, GATA9, GATA12, GATA21, GATA22, GATA23
MADF;Trihelix	GT-1
MYB-related/Myb/SANT	RVE7
NF-YA	NFYA5
NF-YB	NFYB9
	**SA-responsive TFs**
bZIP	TGA1, TGA2, TGA3, TGA5, TGA7
Dof	DOF1.1, DOF3.4, DOF3.6
MYB	MYB46
MYB-related	RVE4, RVE6, RVE8
WRKY	WRKY3, WRKY4, WRKY6, WRKY7, WRKY8 WRKY15, WRKY18, WRKY21, WRKY26, WRKY30, WRKY38, WRKY40, WRKY53, WRKY54, WRKY60, WRKY70
	**bacterium-responsive TFs**
bZIP	TGA3, TGA7
WRKY	WRKY8, WRKY11, WRKY17, WRKY18, WRKY27, WRKY33, WRKY38, WRKY40, WRKY48, WRKY52, WRKY53, WRKY60, WRKY70
	**auxin-responsive TFs**
bZIP	TGA1, TGA2, TGA3, TGA5, TGA7
C2H2	AZF2
Dof	DOF1.1, DOF3.4, DOF3.6
Homeodomain; HB-PHD	PRH
MADS box; MIKC	AGL14, AGL15
MYB-related	RVE4, RVE5, RVE8
WRKY	WRKY23
	**ABA-responsive TFs**
bHLH	SCRM, MYC2
C2H2	AZF2, AZF3, ZAT10
Dehydrin	COR15A
MYB-related	RVE4, RVE5, RVE6, RVE8
NF-YA	NFYA5
NF-YB	NFYB6, NFYB9
NF-YC	NFYC3
WRKY	WRKY8, WRKY25, WRKY33
	**gibberellin-responsive TFs**
MADS box	AGL42, AGL71, AGL72
MYB-related	RVE4, RVE5, RVE6, RVE8
WRKY	WRKY27
	**chitin-responsive TFs**
C2H2	ZAT10
Dof	DOF1.7
WRKY	WRKY6, WRKY11, WRKY17, WRKY18, WRKY22, WRKY33, WRKY40, WRKY46, WRKY48, WRKY53, WRKY70
	**cold-responsive TFs**
bHLH	SCRM
C2H2	AZF2, AZF3, ZAT6, ZAT10
Dehydrin	COR15A

Dof	DOF2.5
MADS box; MIKC	AGL19
WRKY	WRKY25, WRKY33, WRKY34
	**salt stress-responsive TFs**
bHLH	MYC2, SCRM
C2H2	AZF3, ZAT10
Dehydrin	COR15A
MYB-related	RVE3, RVE4, RVE5, RVE6, RVE8
MYB-related/Myb/SANT	RVE7
WRKY	WRKY8, WRKY25, WRKY33, WRKY46, WRKY57
