# Supplementary material for: Isolation and Comprehensive in Silico Characterisation of a New 3-Hydroxy-3-Methylglutaryl-Coenzyme A Reductase 4 (HMGR4) Gene Promoter from Salvia miltiorrhiza: Comparative Analyses of Plant HMGR Promoters
Source: Plants (Basel). 2022 Jul 16;11(14):1861. doi: 10.3390/plants11141861 (PMC9318348; doi:10.3390/plants11141861)
Supplement: Supplementary file 1 [file plants-11-01861-s001.zip › Table S3.pdf]

Table S3. TF genes co-expressed with *A. thaliana HMGR1* found with the Expression Angler tool.

TF locus name	TF gene name	*r*-value [a]
	**AtGenExpress Hormone and Chemical Compendium**	
At5g17300	*RVE1*	0.501
At1g06850	*AtbZIP52*	0.501
At2g01760	*ARR14*	0.509
At1g33240	*GTL1*	0.509
At1g19700	*BLH10*	0.516
At1g64380	*ERF061*	0.516
At5g44190	*GLK2*	0.520
At5g54630	*MRB17.13*	0.521
At1g06040	*BBX24*	0.522
At5g25810	*TINY*	0.527
At3g23050	*IAA7*	0.538
At5g13770	Pentatricopeptide repeat (PPR-like) superfamily protein	0.544
At1g22590	*AGL87*	0.547
At1g69690	*TCP15*	0.549
At2g27050	*EIL1*	0.554
At3g06590	*BHLH148*	0.561
At4g40060	*ATHB-16*	0.562
At5g57660	*COL5*	0.563
At4g36540	*BEE2*	0.566
At4g00150	*SCL6*	0.567
At5g02840	*RVE4*	0.573
At1g30650	*WRKY14*	0.576
At1g13300	*HRS1*	0.578
At3g07650	*COL9*	0.578
At1g69780	*ATHB-13*	0.594
At2g18160	*BZIP2*	0.597
At4g09460	*MYB6*	0.599
At2g42380	*BZIP34*	0.605
At3g05800	*BHLH150*	0.609
At2g28200	*ZAT5*	0.61
At1g74840	Homeodomain-like superfamily protein	0.61
At3g17100	*BHLH147*	0.618
At1g68520	*BBX14*	0.621
At5g47390	*KUA1*	0.623
At3g60490	*ERF035*	0.63
At3g62420	*BZIP53*	0.635
At3g58120	*BZIP61*	0.64
At2g23760	*BLH4*	0.648
At3g48360	*BT2*	0.659
At3g47620	*TCP14*	0.704
At5g60850	*DOF5.4*	0.727
	**AtGenExpress Abiotic Stress Compendium**	
At3g16770	*RAP2-3*	0.502
At5g46690	*BHLH071*	0.502
At1g68190	*BBX27*	0.502
At3g47620	*TCP14*	0.502
At1g69690	*TCP15*	0.503
At2g20570	*GLK1*	0.504
At3g12730	Homeodomain-like superfamily protein	0.506
At3g61150	*HDG1*	0.507

At5g67180	*TOE3*	0.508
At5g44190	*GLK2*	0.512
At2g42300	*BHLH48*	0.518
At5g38140	*NFYC12*	0.523
At4g00730	*ANL2*	0.524
At2g46530	*ARF11*	0.524
At2g02080	*IDD4*	0.525
At3g48590	*NFYC1*	0.527
At4g36870	*BLH2*	0.53
At2g01760	*ARR14*	0.53
At3g17100	*BHLH147*	0.537
At2g22540	*SVP*	0.537
At5g02840	*RVE4*	0.545
At1g72740	Homeodomain-like/winged-helix DNA-binding family protein	0.545
At3g57800	*BHLH60*	0.545
At5g08330	*TCP21*	0.546
At5g05550	*ENAP2*	0.552
At5g24930	*COL4*	0.557
At1g10610	*BHLH90*	0.558
At2g33500	*BBX12*	0.575
At5g08520	*MYBS2*	0.575
At1g54060	*ASIL1*	0.579
At4g21750	*ATML1*	0.583
At4g04890	*PDF2*	0.587
At2g33810	*SPL3*	0.596
At3g19860	*BHLH121*	0.611
At2g43010	*PIF4*	0.625
At1g14920	*GAI*	0.627
At5g62000	*ARF2*	0.63
	**AtGenExpress Pathogen compendium**	
At3g19580	*AZF2*	0.503
At1g69310	*WRKY57*	0.504
At1g02220	*NAC003*	0.505
At1g07530	*SCL14*	0.516
At1g19850	*ARF5*	0.515
At3g01970	*WRKY45*	0.515
At4g39100	*SHL*	0.52
At3g19860	*BHLH121*	0.521
At1g62990	*KNAT7*	0.522
At1g73730	*EIL3*	0.525
At5g52510	*SCL8*	0.526
At2g36080	*ABS2*	0.53
At1g79180	*MYB63*	0.534
At1g48000	*MYB112*	0.535
At2g21240	*BPC4*	0.536
At3g61890	*ATHB-12*	0.536
At5g49700	*AHL17*	0.537
At3g06490	*MYB108*	0.538
At3g51960	*BZIP24*	0.543
At4g27410	*NAC072*	0.543
At2g28200	*ZAT5*	0.547
At1g56010	*NAC021*	0.554
At1g06180	*MYB13*	0.557
At3g17100	*BHLH147*	0.559

At3g58710	*WRKY69*	0.564
At4g31420	*REIL1*	0.565
At2g46680	*ATHB-7*	0.571
At3g20770	*EIN3*	0.572
At5g13330	*RAP2.6L*	0.573
At5g24800	*BZIP9*	0.59
At3g15500	*NAC055*	0.594
At1g10170	*NFXL1*	0.599
At2g38340	*DREB2E*	0.611
At5g39610	*NAC92*	0.643
	**AtGenExpress Tissue Compendium**	
At1g77980	*AGL66*	0.506
At3g15540	*IAA19*	0.512
At2g40620	*BZIP18*	0.517
At1g60240	NAC (No Apical Meristem) domain transcriptional regulator superfamily protein	0.52
At2g03060	*AGL30*	0.521
At3g01470	*HAT5*	0.521
At2g32460	*MYB101*	0.521
At1g72740	Homeodomain-like/winged-helix DNA-binding family protein	0.525
At3g10470	C2H2-type zinc finger family protein	0.534
At1g18750	*AGL65*	0.535
At3g16350	Homeodomain-like superfamily protein	0.539
At2g42380	*BZIP34*	0.546
At4g08250	*SCL26*	0.548
At4g16110	*ARR2*	0.552
At2g47810	*NFYB5*	0.558
At4g14410	*BHLH104*	0.563
At5g56270	*WRKY2*	0.575
At4g35700	*DAZ3*	0.579
At1g35490	bZIP family transcription factor	0.582
At5g45710	*HSFA4C*	0.584
At4g20380	*LSD1*	0.598
At4g31420	*REIL1*	0.609
At1g53320	*TLP7*	0.64
At3g54620	*BZIP25*	0.666
At1g50640	*ERF3*	0.721
	**AtGenExpressPlus Extended Tissue Compendium**	
At4g26930	*MYB97*	0.505
At1g18750	*AGL65*	0.51
At4g35700	*DAZ3*	0.512
At2g23340	*DEAR3*	0.516
At2g01930	*BPC1*	0.519
At5g67580	*TRB2*	0.525
At2g34440	*AGL29*	0.53
At3g20310	*ERF7*	0.534
At2g32460	*MYB101*	0.545
At2g03060	*AGL30*	0.547
At1g35490	bZIP family transcription factor	0.553
At5g56270	*WRKY2*	0.557
At1g34190	*NAC017*	0.558
At3g57390	*AGL18*	0.567
At2g42380	*BZIP34*	0.572
At3g20770	*EIN3*	0.572
At2g40620	*BZIP18*	0.572

At3g16350	Homeodomain-like superfamily protein	0.601
At4g37180	*HHO5*	0.601
At5g46910	*JMJ13*	0.604
At4g16110	*ARR2*	0.611
At5g54680	*BHLH105*	0.64
At4g13980	*HSFA5*	0.644
At1g55520	*TBP2*	0.645
At4g31420	*REIL1*	0.661
At4g20380	*LSD1*	0.665
At1g50640	*ERF3*	0.666
At1g53320	*TLP7*	0.667
At5g45710	*HSFA4C*	0.790

[a] The *r*-value was 0.5 - 1.0.
