# Supplementary material for: Isolation and Comprehensive in Silico Characterisation of a New 3-Hydroxy-3-Methylglutaryl-Coenzyme A Reductase 4 (HMGR4) Gene Promoter from Salvia miltiorrhiza: Comparative Analyses of Plant HMGR Promoters
Source: Plants (Basel). 2022 Jul 16;11(14):1861. doi: 10.3390/plants11141861 (PMC9318348; doi:10.3390/plants11141861)
Supplement: Supplementary file 1 [file plants-11-01861-s001.zip › Table S4.pdf]

Table S4. Primers used in the study.

Primer name	Sequence
**Genome Walker gene-specific primers (GSPs)**	
GSP1	GGTGAAGAAAATGCCGTTGGTTAGGAAGA
GSP2	TGTGGCGGAGTGAGGCGACGGCGGTTT
**Sanger sequencing of *HMGR4* promoter**	
M13_F	GTAAAACGACGGCCAG
M13_R	CAGGAAACAGCTATGAC
HMGR4_F	TTTGTGCAGTGCGAACCAACCAA
HMGR4_R	GCATGACATAGTTCTTTTAGG
